# Donor-Derived Smoldering Multiple Myeloma following a Hematopoietic Cell Transplantation for AML

**DOI:** 10.1155/2017/3728429

**Published:** 2017-02-20

**Authors:** Bita Fakhri, Mark Fiala, Michael Slade, Peter Westervelt, Armin Ghobadi

**Affiliations:** ^1^Washington University School of Medicine, St. Louis, MO, USA; ^2^Siteman Cancer Center, St. Louis, MO, USA

## Abstract

Posttransplant Lymphoproliferative Disorder (PTLD) is one of the most common malignancies complicating solid organ transplantation. In contrast, PTLD accounts for a minority of secondary cancers following allogeneic hematopoietic cell transplantation (HCT). Here we report on a 61-year-old woman who received an ABO-mismatched, HLA-matched unrelated donor hematopoietic cell transplantation from a presumably healthy donor for a diagnosis of acute myeloid leukemia (AML). Eighteen months following her transplant, she developed a monoclonal gammopathy. Bone marrow studies revealed 10% plasma cells, but the patient lacked clinical defining features of multiple myeloma (MM); thus a diagnosis of smoldering multiple myeloma (SMM) was established. Cytogenetic and molecular studies of the bone marrow confirmed the plasma cells were donor-derived. The donor lacks a diagnosis of monoclonal gammopathy of undetermined significance, SMM, or MM.

## 1. Introduction

Infrequently, acute leukemia and lymphoma can develop de novo in engrafted cells of donor origin. Donor cell leukemia/lymphoma (DCL) was first described in 1971 [1], but for many years, the rarity of reported cases made it seem an uncommon occurrence. However, recent advances in molecular chimerism techniques and engraftment analysis suggest that this entity may be significantly more common than previously reported. A growing body of evidence suggests that DCL might represent up to 5% of all posttransplant leukemia relapses [2]. In many cases, the donor has later been diagnosed with the malignancy, indicating inadvertent transfer of tumor/pretumor clonal cells from the donor to the recipient. Instances of spontaneous DCL are less common and highlight Stephen Paget's original “seed and soil” hypothesis [3], indicating the significance of microenvironment in leukemogenesis/lymphomagenesis.

## 2. Case Presentation

An otherwise healthy 61-year-old Caucasian woman presented in 2010 with anemia and thrombocytopenia after one month of recurrent upper respiratory infections. White blood cell (WBC) count at the time of presentation was 8.7 with 48% circulating blasts, hemoglobin was 9.6, and platelets were 39,000. Bone marrow biopsy was consistent with a diagnosis of acute myeloid leukemia (AML) with translocation of chromosomes 11 and 19 involving the MLL locus [t(11:19)(q23; p13.1); MLL-ENL]. She received two rounds of induction therapy with idarubicin and cytarabine with complete response. She then received one cycle of High-Dose Cytarabine (HiDAC) consolidation. She also received 1 cycle of decitabine as there were significant delays in identifying a suitable stem cell donor.

She underwent a matched unrelated donor (MUD) hematopoietic cell transplantation (HCT) with fludarabine, busulfan, and thymoglobulin conditioning seven months following the initial diagnosis from a presumably healthy male donor. Graft versus Host Disease (GvHD) prophylaxis regimen consisted of methotrexate, tacrolimus, and mycophenolate mofetil. Day +30 post-HCT bone marrow showed complete remission with complete donor cell engraftment. Her immediate posttransplant course was complicated by mild acute gastrointestinal GvHD and human polyomavirus (BK) cystitis. Post-HCT bone marrow biopsy on days +100 and +180 again showed complete remission with complete donor cell engraftment. In the absence of any evidence of ongoing GvHD, at 18 months after HCT all immunosuppressive agents were discontinued after a prolonged taper schedule.

Five hundred and sixty days following her transplant, the patient was found to have abnormally high IgG levels during routine follow-up (3080; normal range 700–1450). Serum protein electrophoresis (SPEP) showed two abnormal restricted peaks in gamma region (*a* = 0.1, *b* = 2.4). Serum protein immunofixation classified the protein as IgG lambda monoclonal protein. Additional laboratory work-up was significant for increased lambda free light chain (FLC) (6.54; normal range: 0.57–2.63), normal kappa FLC (1.49; 0.33–1.94), low kappa/lambda FLC ratio (0.23; 0.26–1.65), and a raised beta-2-microglobulin (3.6; normal range: 1.10–2.50). Bone marrow biopsy revealed a normocellular bone marrow with trilineage hematopoiesis with no excess blasts but was significant for infiltrating plasma cells. An immunohistochemical stain for CD138 was performed to better quantify the plasma cells in a tissue context and showed singly dispersed and clustered plasma cells comprising approximately 10% of the marrow cellularity. Flow cytometric analysis showed approximate 1% of total events to be lambda light chain restricted plasma cells that did not express CD56. In situ hybridization for EBV (EBER) on the bone marrow biopsy was performed and was negative. Patient's hemoglobin, calcium level, and kidney function were within normal limits. A bone survey was performed and did not identify any lytic or blastic lesions. To further characterize the origin of plasma cells, CD138+ cells were isolated using a Miltenyi autoMACS device (Miltenyi Biotech) and then short tandem repeat (STR) polymorphic DNA markers were amplified by polymerase chain reaction (PCR), labeled with fluorescent markers, and used to distinguish patient and donor cells as different on a Capillary Electrophoresis Fluorescence Detection instrument. The results were consistent with complete donor cell engraftment (<5% recipient cells present).

Given the presence of monoclonal gammopathy and 10% bone marrow plasma cells in the absence of end-organ damage (lytic lesions, anemia, renal disease, or hypercalcemia) that could be attributed to the underlying plasma cell disorder or other myeloma defining events, a diagnosis of donor-derived smoldering multiple myeloma (SMM) was established. The National Marrow Donor Program (NMDP) was notified. The NMDP has confirmed that the donor does not have a diagnosis of SMM or MM. It is unclear if the donor has ever had a bone marrow biopsy or SPEP to confirm or if he simply lacks the diagnosis.

We continued to observe our patient regularly for changes in her myeloma markers. At the time of preparation of this manuscript, her myeloma markers have been stable for over four years. With regard to AML, she is almost 6 years out of MUD-HCT and has been in remission ever since.

## 3. Discussion

To date very few cases of donor origin PTLD-plasma cell neoplasms have been reported in literature. Three cases have been described with solid organs in kidney and heart-lung transplantation [4, 5]. Peri et al. reported a case of EBV-negative, PTLD-MM in a 67-year-old female, presenting 18 months after kidney transplantation from a male donor. FISH analysis of the tumor revealed a Y chromosome in the majority of the cells, signifying that the neoplasm was derived from the donor kidney [4]. Yousem et al. reported 2 cases of donor origin PTLD who developed monoclonal gammopathy of undetermined significance (MGUS) in a 28-year-old female and an 18-year-old male patient who underwent heart-lung transplantation due to primary pulmonary hypertension [5].

Kim et al. reported a 40-year-old woman with a diagnosis of refractory anemia with ring sideroblast,who underwent an allogeneic HCT from a 32-year-old healthy male donor. Almost four months after HCT, she developed progressive pancytopenia, biclonal gammopathy, and IGH gene rearrangement. A bone marrow biopsy revealed 12% plasma cells with an immunoglobulin heavy-chain gene rearrangement. Cytogenetics and chimerism studies confirmed a diagnosis of donor cell origin PTLD-MM [6]. Upon additional investigation, the donor was deemed to be MM-free. This was the first report of donor-derived MM following HCT in a patient whose donor lacks the diagnosis.

There have been two reported cases of PTLD-MM with possible transfer of the malignant clone from donor to recipient. Kumar et al. reported on a 42-year-old man transplanted for chronic phase chronic myeloid leukemia (CP-CML). The donor was diagnosed with MM 27 months following the stem cell donation; the recipient developed IgA myeloma 40 months after HCT. Serum electrophoresis and bone marrow investigations established a diagnosis of IgA kappa MM in both donor and recipient [7]. Similarly, Maestas et al. reported on a 54-year-old woman with a diagnosis of CML who received a sibling allogeneic HCT from her brother and was diagnosed with donor-derived IgG kappa SMM 12 years later [8]. Her brother was also evaluated and was diagnosed with SMM.

PTLD is a life-threatening complication after HCT or solid organ transplantation. The majority of PTLD is of B-cell origin and associated with Epstein-Barr virus (EBV). During the past decade, progress has been made in better understanding both the pathogenesis of PTLD and early detection strategies [9]. Several studies have tried to ascertain the major risk factors associated with early PTLD [10, 11]. These risk factors include an unrelated or HLA-mismatched related donor [10, 11], T cell depletion of donor marrow [10, 11], administration of anti-thymocyte globulin (ATG) [10, 11] or anti-CD3 antibodies for prophylaxis or treatment of acute GvHD [10], chronic GvHD [10], and age ≥ 50 [11].

Despite elaborate efforts in identifying the risk factors associated with the development of PTLD, the pathogenesis of donor cell origin PTLD after HCT has not been thoroughly understood. Various components in the marrow tightly regulate hematopoietic cell fate. These players include osteoblasts, their mesenchymal precursors, CXCL12 rich reticular cells, and adipocytes. In addition to these cells, hormones such as estrogen, PTH, and catecholamines also contribute to the activation of the microenvironment [12, 13]. The interplay of the abovementioned cells and pathways in determining the fate of stem cell remains to be fully established.

Given the lack of a diagnosis of MGUS/SMM/MM in the donor described in our case, we hypothesized that the tumor microenvironment played a critical role in the development of donor-derived MM in our patient.

## 4. Conclusion

Here we report on a patient who received an ABO-mismatched, MUD-HCT for a diagnosis of AML. Eighteen months after HCT, the patient developed donor origin SMM. To the best of our research to date, the donor lacks a diagnosis of MGUS/SMM/MM. This is the second case report of donor-derived MM following HCT in a patient whose donor lacks the diagnosis.
